# Effect of the Abnormal Expression of BMP-4 in the Blood of Diabetic Patients on the Osteogenic Differentiation Potential of Alveolar BMSCs and the Rescue Effect of Metformin: A Bioinformatics-Based Study

**DOI:** 10.1155/2020/7626215

**Published:** 2020-06-07

**Authors:** Chao Liang, Rongxin Sun, Yifan Xu, Wei Geng, Jun Li

**Affiliations:** ^1^Department of Dental Implant Center, Beijing Stomatological Hospital, School of Stomatology, Capital Medical University, Beijing 100050, China; ^2^Beijing Key Laboratory of Tooth Regeneration and Function Reconstruction, School of Stomatology, Capital Medical University, Beijing 100050, China

## Abstract

The success rate of oral implants is lower in type 2 diabetes mellitus (T2DM) patients than in nondiabetic subjects; functional impairment of bone marrow-derived mesenchymal stem cells (BMSCs) is an important underlying cause. Many factors in the blood can act on BMSCs to regulate their biological functions and influence implant osseointegration, but which factors play important negative roles in T2DM patients is still unclear. This study is aimed at screening differentially expressed genes in the blood from T2DM and nondiabetic patients, identifying which genes impact the osteogenic differentiation potential of alveolar BMSCs in T2DM patients, exploring drug intervention regimens, and providing a basis for improving implant osseointegration. Thus, a whole-blood gene expression microarray dataset (GSE26168) of T2DM patients and nondiabetic controls was analyzed. Based on Gene Ontology (GO) and Kyoto Encyclopedia of Genes and Genomes (KEGG) results, differentially expressed genes and signaling pathways related to BMSC osteogenic differentiation were screened, and major risk genes were extracted based on the mean decrease Gini coefficient calculated using the random forest method. Bone morphogenetic protein-4 (BMP-4), with significantly low expression in T2DM blood, was identified as the most significant factor affecting BMSC osteogenic differentiation potential. Subsequently, metformin, a first-line clinical drug for T2DM treatment, was found to improve the osteogenic differentiation potential of BMSCs from T2DM patients via the BMP-4/Smad/Runx2 signaling pathway. These results demonstrate that low BMP-4 expression in the blood of T2DM patients significantly hinders the osteogenic function of BMSCs and that metformin is effective in counteracting the negative impact of BMP-4 deficiency.

## 1. Introduction

With the continuous development of oral implantology, implant restoration has become the preferred treatment option for patients with dentition defects [1]. However, type 2 diabetes mellitus (T2DM) has long been considered a relative contraindication for oral implant surgery [2, 3]. Although the risk of implant osseointegration failure in diabetic patients has decreased as implant surface treatment techniques improved [4], the healing-stage success rate and long-term survival rate of implants in diabetic patients are still significantly lower than those in nondiabetic patients [5]. Due to the complexity of the jawbone marrow microenvironment in diabetic patients, it is still challenging to clarify the causes and mechanisms of the aforementioned phenomena.

Bone marrow-derived mesenchymal stem cells (BMSCs) in jawbone marrow are adult stem cells that play important roles in implant osseointegration [6]. When an implant is placed into the jawbone, BMSCs begin to assemble around the surface of the implant. After the BMSCs adhere to the implant surface, the osteogenic differentiation process is initiated, and new bone gradually forms with the assistance of cells, blood, and related cytokines [7]. Previous studies have shown that BMSC proliferation, migration, differentiation, and mineralization in T2DM patients are significantly inferior to those in nondiabetic patients [8]. The weakened osteogenic differentiation potential of BMSCs in T2DM patients might be an important reason for the failure of implant osseointegration at the healing stage.

Sufficient blood supply is an indispensable factor in facilitating implant osseointegration [9]. The blood derived from the capillary bed of the bone marrow cavity not only provides the necessary cells and oxygen for bone tissue regeneration but also carries a large number of active proteins and cytokines [10]. Many proteins, including bone morphogenetic proteins (BMPs), vascular endothelial growth factor (VEGF), basic fibroblast growth factor (bFGF), and transforming growth factor-*β* (TGF-*β*), can transport chemical signals to BMSCs by binding to surface receptors, thereby playing an important role in regulation of BMSC functions [11–14]. Among the BMP family, bone morphogenetic protein-4 (BMP-4) was recently shown to play a catalytic role in skeletal development and tooth formation [15, 16]. BMP-4 can bind bone morphogenetic protein receptor 1 (BMPR1) and further activate Smad signaling to affect the osteogenic differentiation of stem cells [17]. Previous studies have found that the blood of T2DM patients, in addition to alterations in sugar concentration, shows significantly different gene expression levels compared with the blood of nondiabetic individuals [18]. However, it remains unclear which differentially expressed genes in the blood significantly affect the osteogenic differentiation of BMSCs and potentially interfere with implant osseointegration in diabetic patients who undergo oral implantation. Furthermore, finding an effective and convenient way to improve the osteogenic differentiation function of BMSCs in T2DM patients is meaningful for decreasing the risk of implant osseointegration failure. The commonly used blood glucose-controlling drug, metformin, has been reported to improve bone metabolism and induce osteoblastic cell differentiation [19]. However, the mechanism of the effect of metformin on BMSCs still needs to be elucidated.

In the present study, bioinformatics analyses were performed using microarray data of whole blood from T2DM patients and nondiabetic controls to search for differentially expressed genes that are closely related to BMSC osteogenic differentiation, with the aim of providing markers for risk assessment prior to implant surgery for T2DM patients. These genes can also be used as intervention drug targets to improve the biological functions of alveolar BMSCs, providing a theoretical basis for drug selection for diabetic patients to control blood sugar and simultaneously promote implant osseointegration. Based on the findings in bioinformatics analyses, we planned to use recombinant human BMP-4 protein (rhBMP-4) and an inhibitor of downstream Smad to confirm the effect of BMP-4 on the biological function of BMSCs, as well as mechanism, in T2DM patients and to further explore whether the commonly used drug metformin is able to reverse the impairment of BMSC osteogenic function caused by the lack of BMP-4 in T2DM patient blood via the BMP/Smad pathway.

## 2. Materials and Methods

### 2.1. Dataset Source and Gene Set Enrichment Analysis

Gene expression microarray datasets of whole blood from T2DM patients and nondiabetic controls were downloaded from the Gene Expression Omnibus (GEO) database (https://www.ncbi.nlm.nih.gov/gds/?term=), with the accession code GSE26168 (type 2 diabetes mellitus: mRNA and miRNA profiling). A total of 24,526 standardized genetic data from nine T2DM patients (T2DM group, GSM532834-GSM532842) and eight nondiabetic controls (CON group, GSM532819-GSM532826) in the dataset were used for subsequent analysis. Based on the original matrix data of the microarray, gene set enrichment analysis (GSEA) was performed using GSEA v2.2.2 software (Broad Institute, USA) to identify the differences in biological functions between the two groups of blood samples. The analysis parameters were set to default values.

### 2.2. Identification of Differentially Expressed Genes

The online analysis tool GEO2R in the GEO website was used to screen differentially expressed genes in this study. The screening criteria were as follows: (1) fold change in upregulation or downregulation greater than 2 and (2) *p* values < 0.05. Origin 2019 software (OriginLab, USA) was used to construct volcano plots to intuitively represent gene expression differences in blood samples from the T2DM group and the CON group. Heat maps and cluster heat maps of differentially expressed genes in the two groups of blood samples were plotted using MeV 4.9.0 software (J. Craig Venter Institute, USA) to assess systematic differences in gene expression.

### 2.3. Gene Ontology Annotation and Kyoto Encyclopedia of Genes and Genomes Pathway Analysis

Gene Ontology (GO) annotation was performed to facilitate an understanding of the functions of differentially expressed genes, including three subontologies: biological process (BP), cellular component (CC), and molecular function (MF). Pathway analysis of differentially expressed genes using the Kyoto Encyclopedia of Genes and Genomes (KEGG) database was employed to determine which biological pathways play an important role in the gene expression differences. The above analysis was conducted using the online tool DAVID v6.8 (https://david.ncifcrf.gov/). Significant GO terms and KEGG pathways were identified using Fisher's exact test, and the false discovery rate (FDR) was used to correct the *p* values. Bubble diagrams were created using the R v3.6.1 tool (https://www.r-project.org), and the pathway maps were generated with the online KEGG tool (https://www.kegg.jp/).

### 2.4. Analysis of Protein-Protein Interactions

To further investigate the functions of differentially expressed genes at the protein level, protein-protein interactions (PPIs) were analyzed using the online tool STRING v11.0 (https://string-db.org/), and network diagrams were generated using Cytoscape v3.7.1 software (National Resource for Network Biology, USA). The correlations among the differentially expressed proteins in each KEGG pathway were analyzed using the ClueGO v2.5.4 plug-in in Cytoscape v3.7.1 software. Venn diagrams were created using Origin 2019 software (OriginLab, USA) to reveal the number of overlapping proteins shared by each KEGG pathway.

### 2.5. Risk Gene Extraction Based on Random Forest Analysis

The random forest (RF) method is an integrated classifier composed of many decision trees, and each tree depends on the values of a random vector sampled independently [20]. In this study, differentially expressed genes were subjected to RF analysis using the R v3.6.1 tool (https://www.r-project.org) and ranked based on the mean decrease Gini (MDG) coefficient. MDG was used to quantify the contribution of the difference in the expression of each gene to the overall difference between the two groups [21], and comparison of MDGs provided a basis for extraction of the major risk genes.

### 2.6. Clinical Specimen Collection and Primary BMSC Culture

In this study, human alveolar BMSCs were isolated from wasted bone debris from the implant sockets of patients who underwent oral implantation. An informed consent form for the study was signed by each patient before surgery. The study was approved by the Ethics Committee of Beijing Stomatological Hospital, Capital Medical University (ethics approval: CMUSH-IRB-KJ-PJ-2017-01), and was performed in accordance with the ethical standards laid down in the 1964 Declaration of Helsinki and its later amendments.

The primary BMSC culture method used in this study was similar to that described in our previous study [22]. During oral implant surgery, the implant sockets were prepared using a low-speed drilling technique (50 rpm, without irrigation), and the bone debris was then collected from the drill and placed in sterile tubes containing 0.5 ml phosphate-buffered saline (PBS) (HyClone, USA). An electronic balance (Sartorius, Germany) was used to weigh the tubes before and after the addition of the bone debris to enable quantification. After centrifugation, the bone debris was transferred into a 60 mm Petri dish (Corning, USA) with 5 ml of mesenchymal stem cell medium (MSCM) (ScienCell, USA) and placed in a 37°C and 5% CO_2_ incubator for 7 d. Thereafter, the medium was replaced every 3 d.

### 2.7. Flow Cytometric Analysis

Cells were cultured in 60 mm Petri dishes (Corning, USA) at a density of 10^6^ cells/dish overnight and then fixed with 80% methanol. Primary anti-CD34, anti-CD44, anti-CD45, and anti-CD146 rabbit monoclonal antibodies (Abcam, UK) were incubated with the cells at a concentration of 1 *μ*g/10^6^ cells for 30 min. The samples were next incubated using a goat anti-rabbit fluorescent secondary antibody (ABclonal, China) at a concentration of 1 *μ*g/10^6^ cells for 1 h and assessed using FACSCalibur flow cytometry (BD Biosciences, USA).

### 2.8. Enzyme-Linked Immunosorbent Assay

After the bone debris samples of T2DM and nondiabetic patients were centrifuged, the supernatant in the centrifuge tube was collected. The bone morphogenetic protein-4 (BMP-4) content in the bone debris matrix was determined using an enzyme-linked immunosorbent assay (ELISA) kit (R&D Systems, USA) according to the manufacturer's protocol and was quantified per 100 mg of wet bone tissue for comparison.

### 2.9. Alkaline Phosphatase Assay and Alizarin Red S Staining

BMSCs were induced and cultured in osteogenic medium according to a StemPro Osteogenesis Differentiation Kit (Invitrogen, USA). Recombinant human BMP-4 protein (rhBMP-4) (R&D Systems, USA), a Smad inhibitor (LDN-193189) (Selleck, USA), and metformin (TargetMol, USA) were separately added to 0.5 ml medium in 24-well dishes (Corning, USA), and the final concentrations of each substance in the medium were as follows: rhBMP-4 (+: 10 ng/ml; ++: 50 ng/ml), LDN-193189 (+: 0.1 *μ*M; ++: 0.5 *μ*M), and metformin (+: 30 *μ*M; ++: 100 *μ*M). After 10 d of induction, cells were fixed in 70% ethanol for 1 h and stained using an ALP staining kit (Beyotime, China) according to the manufacturer's protocol. Intracellular ALP activity assays of BMSCs were performed at 3, 5, and 7 d of induction using an ALP Activity Assay Kit (Nanjing Jiancheng Bioengineering Institute, China) according to the manufacturer's protocol and were standardized based on protein concentration. After induction for 21 d, cells were fixed in 70% ethanol and stained with 2% alizarin red S staining solution (Sigma-Aldrich, USA) for 5 min. Then, 1 ml of isopropanol was added into each well to dissolve the red perylenequinone derivatives in the calcium nodules, and the optical density (OD) values were measured at a wavelength of 550 nm.

### 2.10. Western Blotting

Cells were lysed in radioimmunoprecipitation assay (RIPA) buffer with 1 : 100 phenylmethylsulfonyl fluoride (PMSF) and 1 : 100 protease inhibitor cocktail (PIC) (Sigma-Aldrich, USA). Protein concentrations were determined using a Bicinchoninic Acid (BCA) Protein Quantitation Kit (Beyotime, China). Protein samples were separated using a premade 15% sodium dodecyl sulfate (SDS) polyacrylamide gel (Bio-Rad, USA) and transferred to polyvinylidene fluoride (PVDF) membranes (Bio-Rad, USA) using a semidry transfer unit (Bio-Rad, USA). The membranes were blocked in 5% nonfat dry milk (Bio-Rad, USA) for 1 h. Primary antibodies were diluted to the recommended concentration in accordance with the manufacturer's instructions and then incubated with the membranes at 4°C overnight. Subsequently, the membranes were incubated with horseradish peroxidase-labeled anti-rabbit secondary antibody (ABclonal, China) for 1 h. Then, the membranes were immersed in electrochemiluminescence (ECL) solution (Bio-Rad, USA) for 3 min and imaged using a ChemiDoc MP Imaging System (Bio-Rad, USA). The primary antibodies included rabbit monoclonal anti-p-Smad1/5/8, anti-Smad1, and anti-Runx2 (Cell Signaling Technology, USA) and rabbit monoclonal anti-*β*-actin (ABclonal, China).

### 2.11. Statistical Analysis

SPSS 23.0 software was used for statistical analyses. All the data were acquired from at least three independent experiments. The data are expressed as the mean ± standard deviation (SD). Student's *t*-test or one-way analysis of variance (ANOVA) was used to determine statistical significance. The level of significance was defined by two *p* values (^∗^*p* < 0.05 and ^∗∗^*p* < 0.01).

## 3. Results

### 3.1. Differentially Expressed Genes and Gene Set Enrichment in the Blood Samples from T2DM Patients and Nondiabetic Controls

**Figure 1:**
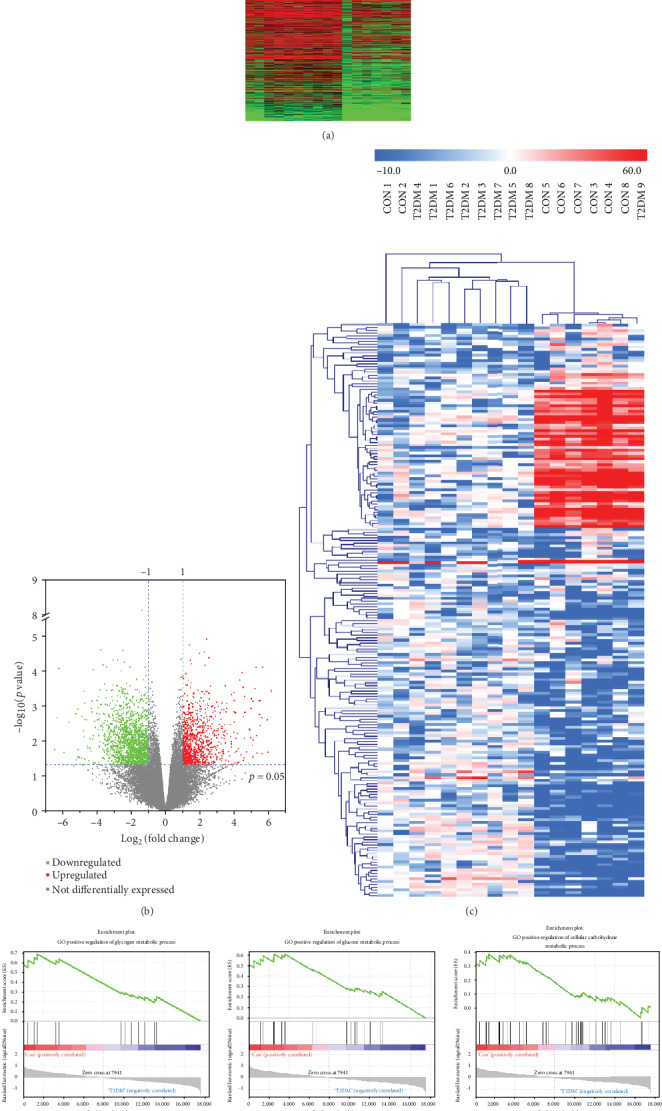
Gene expression differences in the blood from T2DM patients and nondiabetic controls and gene set enrichment analysis (GSEA). (a, b) Heat map and volcano plots showing significantly differentially expressed genes with a greater than twofold change in the blood from the two groups of patients. (c) Cluster heat map showing differentially expressed genes with a fold change greater than 10. (d) GSEA results showing the differences in glucose and lipid metabolism in the blood from T2DM patients and nondiabetic controls.

Heat map and volcano plots showed the genes differentially expressed with a fold change greater than 2 (*p* < 0.05) in blood samples from the T2DM group and the CON group (Figures 1(a) and 1(b)). A cluster heat map showed gene expression profiles with a fold change greater than 10 (*p* < 0.05) in the two groups (Figure 1(c)). In total, 2613 mRNAs were differentially expressed. Among them, 1032 mRNAs were upregulated, and 1581 mRNAs were downregulated in the T2DM group. In particular, 88 mRNAs were upregulated by more than 10-fold, and 117 mRNAs were downregulated by more than 10-fold.

GSEA was conducted for the matrix data of all 24,526 genes in the T2DM and CON groups. The results showed that glycogen metabolic process, glucose metabolic process, cellular carbohydrate metabolic process, fatty acid transport, and lipid catabolic process were significantly downregulated in the T2DM group, and fatty acid biosynthetic process was stronger in the T2DM group than in the CON group (Figure 1(d)). The above results are consistent with the clinical features and pathological manifestations of T2DM.

### 3.2. Functional Analysis of Differentially Expressed Genes

**Figure 2:**
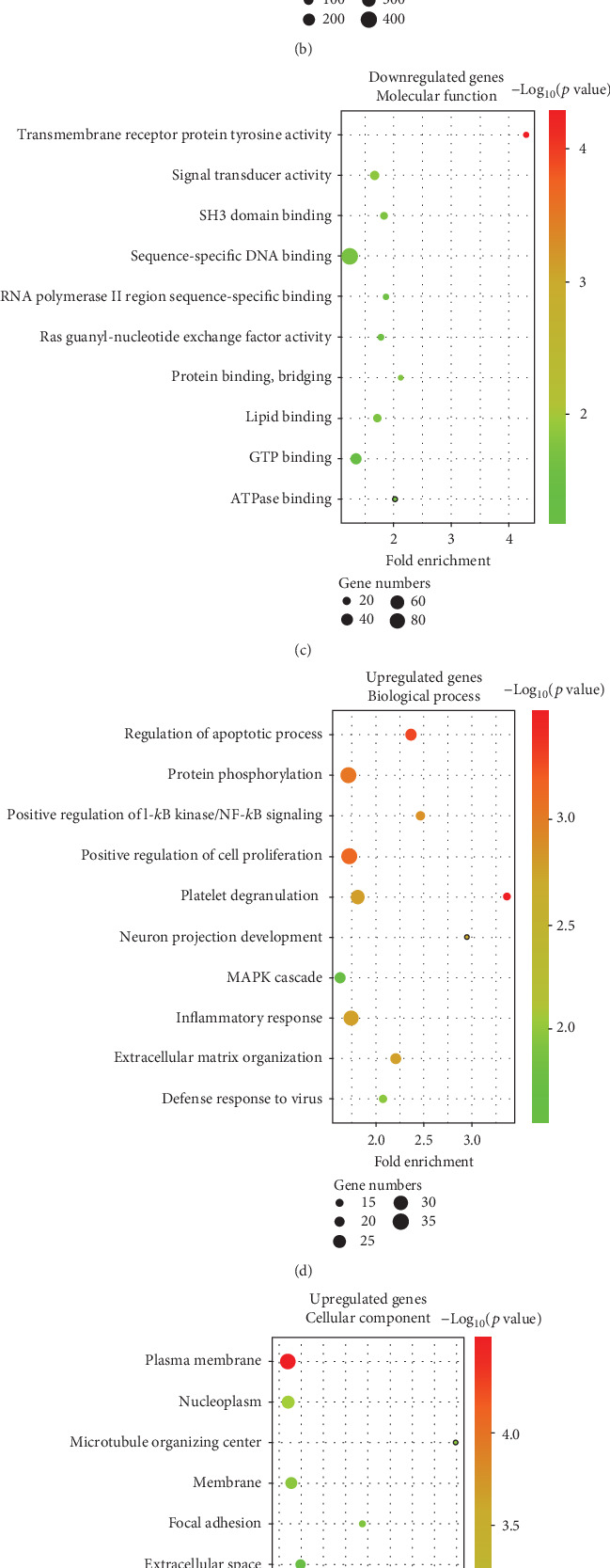
GO annotation of differentially expressed genes in the blood from T2DM patients and nondiabetic controls. Bubble diagrams showing the fold enrichment, gene numbers, and *p* value in the biological process, cellular component, and molecular function of (a–c) downregulated and (d–f) upregulated genes in blood samples from T2DM patients compared with nondiabetic controls.

GO analysis of differentially expressed genes helped identify the differences in blood functions between T2DM patients and nondiabetic individuals. The results showed enrichment of downregulated (Figures 2(a)–2(c)) and upregulated (Figures 2(d)–2(f)) differentially expressed genes in three subontologies: BP, CC, and MF. To explore the possible influences of abnormally expressed factors in the blood of T2DM patients on the osteogenic differentiation potential of BMSCs, we focused on the terms associated with this type of biological function. In the BP enrichment results of downregulated genes in T2DM patients, the fold enrichment of the term “positive regulation of osteoblast differentiation” was 2.49 (11 genes, *p* = 0.01). Follow-up analysis focused on the functions and effects of these 11 genes.

### 3.3. Screening of Key Genes That Regulate Osteoblast Differentiation

**Figure 3:**
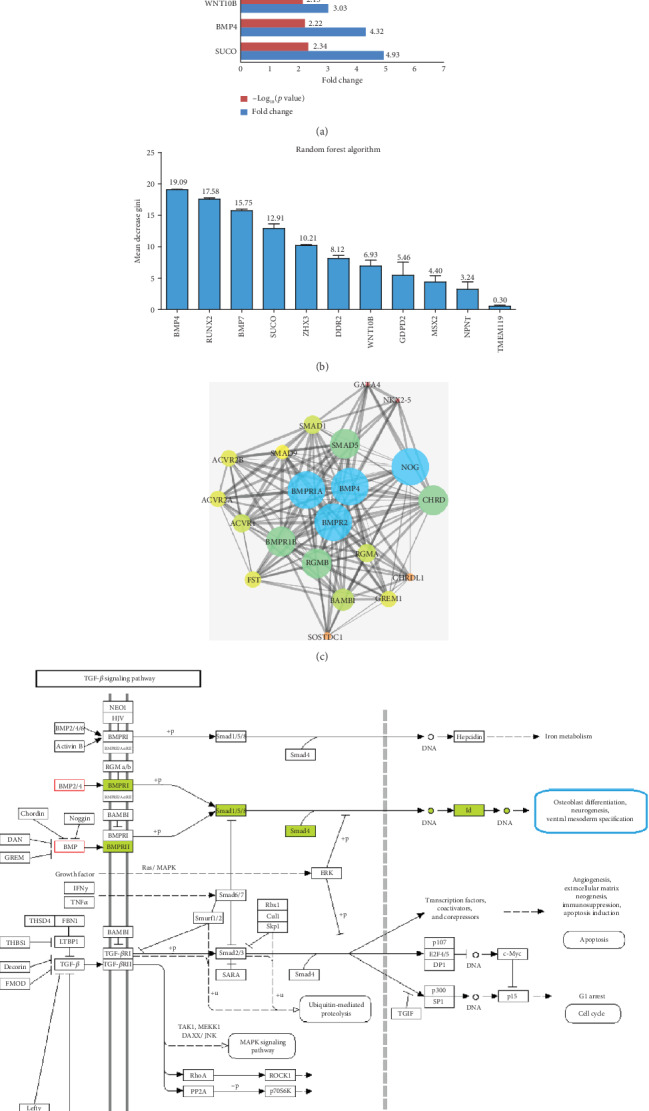
Differentially expressed genes associated with regulation of osteoblast differentiation and major risk gene screening. (a) Fold changes in downregulated genes enriched in the “positive regulation of osteoblast differentiation” term in blood samples from the T2DM group compared with the CON group. (b) The results of random forest analysis showing that BMP-4 has the highest mean decrease Gini coefficient and plays the most important role in “positive regulation of osteoblast differentiation.” (c) Protein-protein interaction analysis of BMP-4 biological function-related proteins showing that BMP-4 is closely related to Smad signaling. (d) Schematic diagram of the TGF-*β* signaling pathway showing that BMP-4 can gradually regulate cell biological functions, such as osteoblast differentiation, by activating Smad signaling.

The expression levels of the 11 genes that could regulate osteoblast differentiation in the blood were significantly higher in the CON group than in the T2DM group. The fold change and *p* value are shown in Figure 3(a). The RF analysis results showed that among the 11 genes, the MDG coefficient for BMP-4 was the highest (19.09), followed by RUNX2 (17.58) and BMP-7 (15.75) (Figure 3(b)). PPI analysis of BMP-4 showed that BMP-4 was closely associated with the Smad signaling proteins (Figure 3(c)). The KEGG pathway map of the TGF-*β* signaling pathway showed that BMP-4 can activate Smad1/5/8 phosphorylation by binding to the cell membrane receptors BMPR1 and BMPR2, thereby promoting osteoblast differentiation and other biological functions by activating IDs for regulation of DNA transcription (Figure 3(d)).

### 3.4. Analysis of Biological Pathways of Differentially Expressed Genes

**Figure 4:**
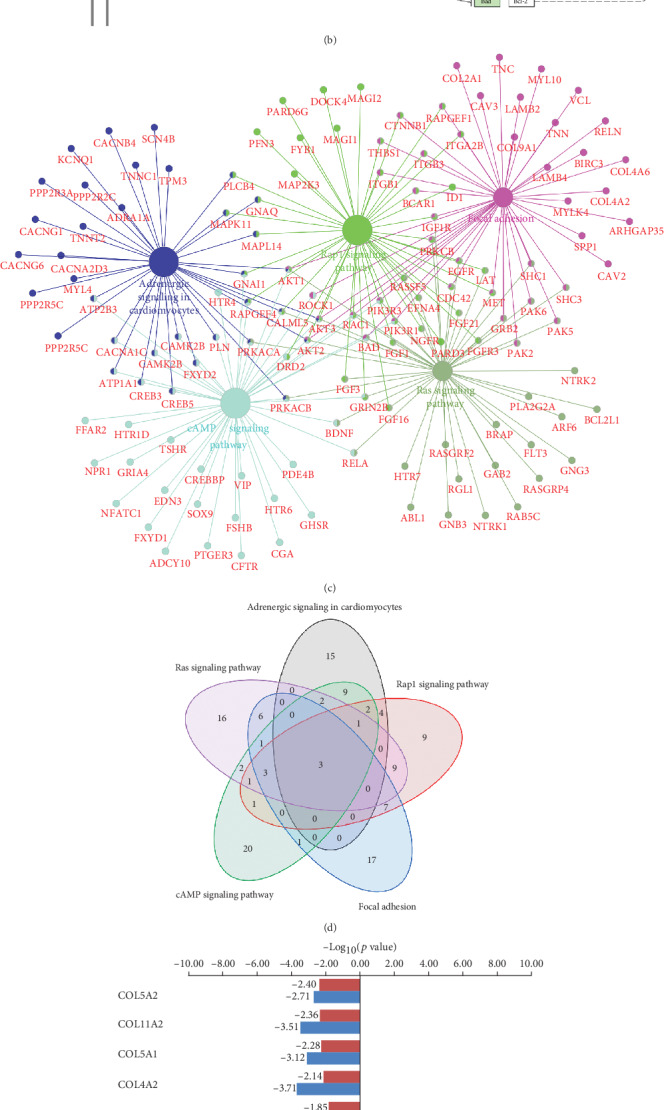
Analysis of biological pathways of differentially expressed genes in the blood of T2DM patients compared with nondiabetic controls. (a) The fold enrichment, gene numbers, and *p* value in KEGG pathway analysis of differentially expressed genes. (b) Schematic diagram of the focal adhesion signaling pathway showing that extracellular matrix proteins can regulate osteoblast differentiation by binding to integrins, increasing FAK phosphorylation, and activating the downstream Wnt signaling pathway. (c) Interaction analysis of differentially expressed genes in the differential KEGG pathways. (d) Venn diagram showing the number of overlapping genes among the KEGG pathways. (e) Differential expression status of the focal adhesion pathway-related collagen genes among the differentially expressed genes (the T2DM group compared with the CON group). (f) Random forest analysis of the aforementioned collagen genes.

Figure 4(a) shows the KEGG pathway analysis results for differentially expressed genes in the blood from the T2DM group compared with that from the CON group. Among them, the focal adhesion pathway plays a critical role in the process of BMSC adhesion to the surface of biomaterials. A total of 45 genes were enriched in the focal adhesion pathway, with a fold enrichment of 1.72 (*p* < 0.01). In the focal adhesion pathway, extracellular matrix proteins can bind to integrins to activate the downstream Wnt signaling pathway and regulate the osteogenic differentiation ability of BMSCs (Figure 4(b)). The interaction analysis among differentially expressed genes in these KEGG pathways showed that the differentially expressed genes in the focal adhesion pathway have associations with the Ras signaling pathway, cAMP signaling pathway, Rap1 signaling pathway, and adrenergic signaling in cardiomyocytes (Figure 4(c)). The Venn diagram in Figure 4(d) shows the number of overlapping genes in these five important pathways.

Collagen represents an important class of extracellular matrix proteins and plays a key role in integrin binding and regulation of focal adhesion-associated signaling pathways. Among the 11 collagens associated with the focal adhesion pathway in the differentially expressed genes, nine were significantly downregulated in the T2DM group (Figure 4(e)), which could negatively affect the adhesion process of BMSCs. RF analysis showed that among the nine genes, COL5A1 had the highest MDG coefficient (29.06), followed by COL9A1 (19.93) and COL2A1 (19.61) (Figure 4(f)).

### 3.5. BMP-4 Expression in the Matrix of Alveolar Bone Debris from Patients Who Underwent Oral Implant Surgery

**Figure 5:**
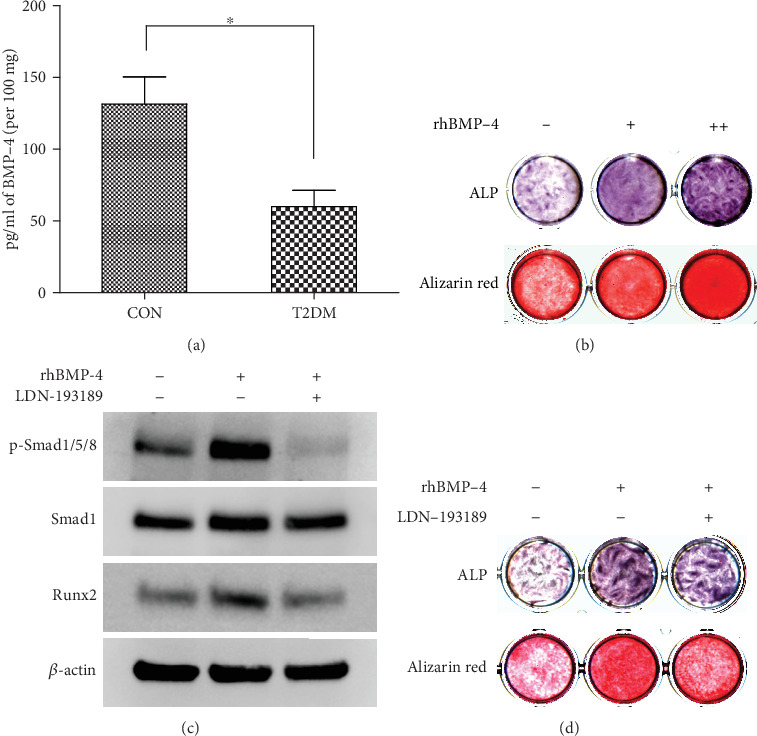
BMP-4/Smad/Runx2 signaling promotes the osteogenic differentiation of BMSCs from T2DM patients. (a) BMP-4 levels in the matrix supernatant of alveolar bone debris from implant sockets of T2DM patients who underwent oral implantation were significantly lower than those of nondiabetic patients. (b) rhBMP-4 dose-dependently enhanced ALP expression and mineralization in BMSCs from T2DM patients; “+” represents 10 ng/ml, and “++” represents 50 ng/ml. (c) Western blotting results showing that rhBMP-4 promoted Runx2 expression by stimulating p-Smad1/5/8 phosphorylation and that LDN-193189 significantly inhibited the aforementioned effects of rhBMP-4. (d) The promoting effect of rhBMP-4 on ALP expression and in vitro mineralization in BMSCs from T2DM patients can be inhibited by LDN-193189. The data are presented as the mean ± standard deviation. ^∗^*p* < 0.05; ^∗∗^*p* < 0.01.

The major components of the alveolar bone debris supernatant samples after centrifugation represented diluted blood of diabetic and nondiabetic patients. Standardized ELISA results showed that the BMP-4 concentration in the matrix supernatant of alveolar bone debris from T2DM patients was significantly lower than that from nondiabetic patients (*p* < 0.05) (Figure 5(a)).

### 3.6. Identification of Human BMSCs

Based on flow cytometric analysis, the cells expressed CD44 (positivity rate 98.2%) and CD146 (positivity rate 98.5%) but did not express CD34 (positivity rate 2.16%) or CD45 (positivity rate 1.06%) (Figure S1a), indicating that the cells express the surface markers of BMSCs. Alizarin red S staining results showed positive calcium node staining after 21 d of osteogenic induction (Figure S1b); this indicated that the cells had osteogenic differentiation potential. These findings confirmed that the cells obtained via the low-speed drilling technique exhibited the characteristics of BMSCs.

### 3.7. BMP-4/Smad/Runx2 Signaling Promotes Osteogenic Differentiation of BMSCs from T2DM Patients

ALP and alizarin red S staining showed that rhBMP-4 could increase ALP expression and the degree of mineralization in BMSCs from T2DM patients, and the effect of 50 ng/ml was more significant than the effect of 10 ng/ml (Figure 5(b)). According to the KEGG pathway map (Figure 3(d)), western blotting was used to detect changes induced in Smad signaling by rhBMP-4. The results showed that rhBMP-4 increased the phosphorylation level of Smad1/5/8 and further promoted Runx2 expression, and the Smad inhibitor LDN-193189 significantly inhibited the above functions of rhBMP-4 (Figure 5(c)). Furthermore, rhBMP-4 was confirmed to regulate ALP expression and in vitro mineralization of BMSCs via the above signaling pathways (Figure 5(d)).

### 3.8. Metformin Improves the Reduced Osteogenic Differentiation Ability of BMSCs from T2DM Patients Caused by Insufficient BMP-4 Combination

**Figure 6:**
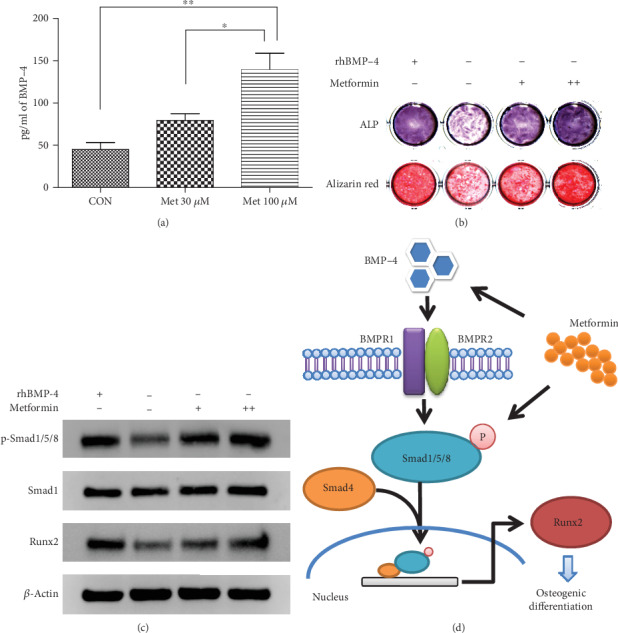
Metformin improves the osteogenic differentiation potential of BMSCs from T2DM patients. (a) Metformin dose-dependently promoted the secretion of BMP-4 by BMSCs from T2DM patients. (b) Western blotting results showing that metformin promoted Runx2 expression by stimulating Smad1/5/8 phosphorylation; “+” represents 30 *μ*M, and “++” represents 100 *μ*M. The effect of 100 *μ*M metformin on Smad signaling activation was similar to that of 50 ng/ml rhBMP-4. (c) Metformin promoted ALP expression and in vitro mineralization in BMSCs from T2DM patients. Again, the effect of 100 *μ*M metformin was similar to that of 50 ng/ml rhBMP-4. (d) Metformin regulates the osteogenic differentiation potential of BMSCs from T2DM patients through the BMP-4/Smad/Runx2 signaling pathway. The data are presented as the mean ± standard deviation. ^∗^*p* < 0.05; ^∗∗^*p* < 0.01.

ELISA results showed that 30 *μ*M metformin promoted BMP-4 secretion by BMSCs from T2DM patients (*p* < 0.05), and the promoting effect of 100 *μ*M metformin was more significant (*p* < 0.01) (Figure 6(a)). Western blotting results showed that metformin increased the Smad1/5/8 phosphorylation level and thus Runx2 expression in BMSCs and that the activation effect of 100 *μ*M metformin on the Smad signaling was similar to that of 50 ng/ml rhBMP-4 (Figure 6(b)). ALP and alizarin red S staining showed that metformin increased the osteogenic differentiation potential of BMSCs from T2DM patients. The effect of 100 *μ*M metformin was more prominent than that of 30 *μ*M, leading to an increase in BMSC ALP expression and in vitro mineralization to a similar extent as that induced by 50 ng/ml rhBMP-4 (Figure 6(c)). In summary, metformin improved the osteogenic differentiation potential of BMSCs from T2DM patients through the BMP-4/Smad/Runx2 signaling pathway (Figure 6(d)).

## 4. Discussion

T2DM is a metabolic disease mainly characterized by hyperglycemia caused by islet dysfunction [23]. Clinically, the risk of oral implant failure in patients with T2DM is significantly higher than that in nondiabetic patients [2, 3, 5]. Abnormal BMSC biological function is an important cause of poor implant osseointegration [24]. Studies have shown that high glucose induction can significantly reduce the biological functions of human alveolar BMSCs, including proliferation, migration, differentiation, and mineralization [25, 26]. However, the etiology and pathology of diabetes are extremely complex, and hyperglycemia is only one of the pathological manifestations of T2DM. Recent clinical studies have also found that the stability of implants during the healing stage in T2DM patients with good glycemic control was still lower than that in nondiabetic patients [27], which indicated that in addition to blood glucose, other important factors exist that can seriously affect implant osseointegration in diabetic patients. Unfortunately, because these factors are still undetermined, there is currently no ideal treatment method to improve implant osseointegration in diabetic patients. In addition, recent meta-analyses and cohort studies have shown that T2DM is strongly associated with increased fracture risk [28]; T2DM is also considered a potential cause of secondary osteoporosis in the population [29]. The reduction in the osteogenic differentiation potential of BMSCs in diabetic patients has been reported to play a key role in reducing bone formation, impairing bone fracture healing, and increasing the degree of osteoporosis [30, 31]. Furthermore, osteoporosis is considered a potential risk factor for oral implant failure [32]. Thus, effectively improving the biological function of BMSCs is beneficial not only for oral implant treatment but also for curing other osteogenic-related health problems in T2DM patients.

Previous studies have shown that many proteins and cytokines in the blood can play key regulatory roles in the biological functions of BMSCs [33]. For example, after BMP family members bind to their receptors on the cell surface, they can regulate osteogenic differentiation of BMSCs via the TGF-*β* signaling pathway [11, 34]; after COL family members bind integrins on the cell membrane, they can regulate the adhesion function of BMSCs via reconstruction of focal adhesions and cytoskeletons [7, 35]. For patients who undergo oral implantation, an abnormal content of the abovementioned cytokines in the blood will inevitably affect multiple biological functions of BMSCs, resulting in unstable implant osseointegration during the healing stage. In the present study, microarray data of blood samples from T2DM patients and nondiabetic controls (dataset GSE26168) were analyzed, the expression levels of many genes in these two groups were found to be significantly different, and the biological functions of a portion of the differentially expressed genes were enriched in osteogenic differentiation. Previous studies have also shown that osteogenic differentiation of alveolar BMSCs is a key step in the implant osseointegration process [6, 24]. Thus, we speculated that in addition to the changes in blood glucose levels, differences in the content of osteogenic differentiation-associated cytokines in the blood may be an important reason for the lower degree of implant osseointegration in T2DM patients than that in nondiabetic patients. However, the reason for such differences in the content of osteogenic differentiation-related cytokines in the blood between T2DM patients and normal controls is still unclear. These cytokines, such as the BMP family, can be synthesized and secreted into the circulation by various tissues and their cells, such as the bone marrow, adipose tissue, kidney, and liver [36–38]. Insulin resistance in T2DM and decreased insulin stimulation can impact metabolism in these tissues [39–41], which may affect the contents of the cytokines released by these tissues into the blood. However, this hypothesis needs to be confirmed by solid experimental research.

In this study, according to the comprehensive analysis of GO annotation and RF results, we found that the difference in the BMP-4 expression level in the blood of T2DM patients and nondiabetic controls may be a major risk factor for the difference in BMSC osteogenic differentiation between these two groups. The expression level of BMP-4 in the T2DM group was significantly lower than that in the CON group. Additionally, we examined the BMP-4 concentration in the matrix supernatant of alveolar bone debris from the implant sockets of T2DM and nondiabetic patients undergoing oral implant surgery, and the results also confirmed a significantly lower BMP-4 concentration in the T2DM group. BMP-4, an important secreted protein in the TGF-*β* superfamily, is widely known for its active functions in embryonic development and formation of the heart and other organs [42]. In recent years, BMP-4 has been considered a necessary regulatory factor of tooth development and bone tissue formation [43]. Binding of BMP-4 to the membrane receptor BMPR1 leads to phosphorylation of the intracellular signal transduction protein Smad1/5/8. p-Smad1/5/8 and Smad4 oligomerize to form a complex, which is transported into the nucleus to act as a transcription factor. Then, the expression of Runx2 and other osteogenic factors can be stimulated [44, 45]. In this study, we also found that rhBMP-4 had a significant promoting effect on osteogenic differentiation of BMSCs from T2DM patients, and this effect was achieved via the BMP-4/Smad/Runx2 axis. Therefore, the reduction in BMP-4 expression in the blood and bone matrix of T2DM patients is bound to affect the osteogenic differentiation potential of BMSCs and likely interferes with implant osseointegration during the healing stage.

Metformin is currently a commonly used and highly effective drug for the treatment of T2DM [46]. Metformin can reduce blood glucose levels by increasing peripheral glucose utilization, reducing intestinal absorption of glucose, and inhibiting hepatic gluconeogenesis [47]. Clinical studies have demonstrated that metformin has protective effects on bone tissue and can decrease the fracture risk in T2DM patients [48]. Metformin also has a potential effect on promoting osteogenic differentiation of BMSCs and preosteoblasts [49], and it enhances ALP expression and the in vitro mineralization ability of osteoblasts [50]. In vivo experiments have also shown that metformin can promote fracture healing and induce new bone formation in rats [51]. Studies on the mechanisms have reported that the ability of metformin to impact osteogenic differentiation is mainly caused via AMP-activated protein kinase (AMPK) signaling [52], whereby phosphorylated AMPK regulates expression of Runx2 to further promote osteogenic function in preosteoblasts [53]. In addition, Runx2 has been widely proven to be regulated by the BMP/Smad pathway in BMSCs [54], but whether metformin promotes Smad signaling is still unclear.

In the present study, we found that metformin could stimulate BMP-4 secretion by BMSCs from T2DM patients. Moreover, metformin was confirmed to activate the downstream target gene Runx2 via Smad signaling to enhance the osteogenic differentiation ability of BMSCs, and the results showed that 100 *μ*M metformin had a promoting effect similar to that of rhBMP-4. Therefore, whether through stimulation of BMP-4 secretion or direct promotion of osteogenic differentiation via Smad/Runx2, metformin could compensate for the negative impact of low blood BMP-4 levels on the osteogenic potential of BMSCs in diabetic patients. These findings demonstrate another important application value of metformin in T2DM patients who undergo oral implantation: not only can the blood glucose levels be controlled by metformin, but it also has potential effect to improve implant osseointegration during the healing stage by enhancing the osteogenic differentiation of alveolar BMSCs.

In addition, based on the bioinformatics analysis results in this study, we found that a portion of the specific extracellular matrix (ECM) proteins in the blood, especially collagens, may play important roles in the process of BMSC-implant adhesion [9, 43]. Previous studies have shown that BMSC adhesion onto an implant surface is the initial step that precedes their subsequent functions [55]. After a biomaterial is implanted into the bone tissue, collagens in the blood will rapidly adsorb onto the surface of the biomaterial. Then, BMSCs recognize the tripeptide sequence Arg-Gly-Asp (RGD) in collagens through integrins, which promotes cell adherence onto the biomaterial surface and initiates subsequent cell spreading, proliferation, and osteogenic differentiation [56, 57]. In the present study, KEGG pathway analysis showed that the expression of focal adhesion signaling pathway-related genes was significantly different in the blood from T2DM patients and nondiabetic controls and that the expression of most collagen mRNAs was low in the T2DM group. The above results provide new ideas for future exploration of how to improve implant osseointegration in diabetic patients who undergo oral implantation. Increasing the adsorption of collagens by modifying the implant surface or using molecular agents to improve the adhesion ability of BMSCs in T2DM patients is an important direction for future research.

## 5. Conclusions

In summary, significant differences exist in the gene expression levels in the blood from T2DM patients and nondiabetic individuals. The low BMP-4 expression level in the blood from T2DM patients can affect the osteogenic differentiation potential of alveolar BMSCs. Metformin, a first-line clinical drug for treatment of T2DM, can significantly improve the osteogenic differentiation of BMSCs via the BMP-4/Smad/Runx2 signaling pathway, thereby compensating for the negative impact of BMP-4 deficiency on the functions of BMSCs from T2DM patients.

## Data Availability

The data used to support the findings of this study are available from the corresponding author upon request.

## Supplementary Materials

Supplementary Materials Figure S1: identification of human BMSCs. (a) Flow cytometry analysis showed that the cells expressed CD44 and CD146 but did not express CD34 or CD45. (b) Alizarin red staining was positive after 21 d of osteogenic induction. These findings confirmed that the cells have characteristics consistent with those of BMSCs.
