# Supplementary material for: Effect of the Abnormal Expression of BMP-4 in the Blood of Diabetic Patients on the Osteogenic Differentiation Potential of Alveolar BMSCs and the Rescue Effect of Metformin: A Bioinformatics-Based Study
Source: Biomed Res Int. 2020 Jun 7;2020:7626215. doi: 10.1155/2020/7626215 (PMC7298258; doi:10.1155/2020/7626215)
Supplement: Supplementary Materials — Figure S1: identification of human BMSCs. (a) Flow cytometry analysis showed that the cells expressed CD44 and CD146 but did not express CD34 or CD45. (b) Alizarin red staining was positive after 21 d of osteogenic induction. These findings confirmed that the cells have characteristics consistent with those of BMSCs. [file 7626215.f1.pdf]

Supplementary Materials

Figure S1:

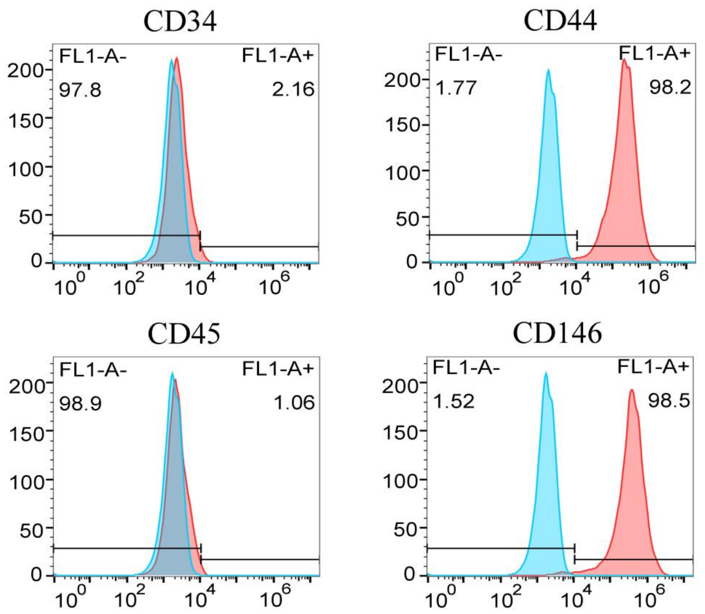

(a)

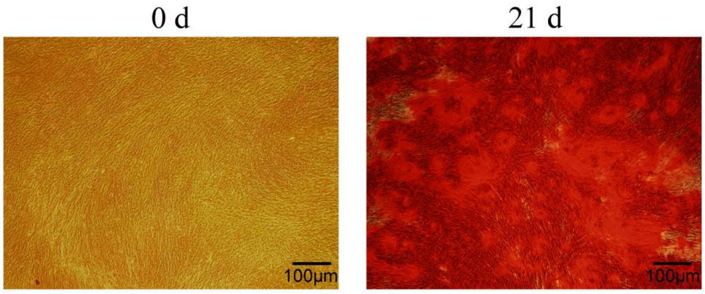

(b)

Figure S1: Identification of human BMSCs. (a) Flow cytometry analysis showed that the cells expressed CD44 and CD146 but did not express CD34 or CD45. (b) Alizarin red staining was positive after 21 d of osteogenic induction. These findings confirmed that the cells have characteristics consistent with those of BMSCs.
